# The Emerging Role of the Lysosome in Parkinson’s Disease

**DOI:** 10.3390/cells9112399

**Published:** 2020-11-02

**Authors:** Alba Navarro-Romero, Marta Montpeyó, Marta Martinez-Vicente

**Affiliations:** 1 Neurodegenerative Diseases Research Group, Vall d’Hebron Research Institute-CIBERNED, 08035 Barcelona, Spain; alba.navarro@vhir.org (A.N.-R.); marta.montpeyo@vhir.org (M.M.); 2 Autonomous University of Barcelona, 08193 Barcelona, Spain

**Keywords:** Parkinson’s disease, lysosomes, autophagy, lysosomal storage diseases, endocytosis, glucocerebrosidase, α-synuclein

## Abstract

Lysosomal function has a central role in maintaining neuronal homeostasis, and, accordingly, lysosomal dysfunction has been linked to neurodegeneration and particularly to Parkinson’s disease (PD). Lysosomes are the converging step where the substrates delivered by autophagy and endocytosis are degraded in order to recycle their primary components to rebuild new macromolecules. Genetic studies have revealed the important link between the lysosomal function and PD; several of the autosomal dominant and recessive genes associated with PD as well as several genetic risk factors encode for lysosomal, autophagic, and endosomal proteins. Mutations in these PD-associated genes can cause lysosomal dysfunction, and since α-synuclein degradation is mostly lysosomal-dependent, among other consequences, lysosomal impairment can affect α-synuclein turnover, contributing to increase its intracellular levels and therefore promoting its accumulation and aggregation. Recent studies have also highlighted the bidirectional link between Parkinson’s disease and lysosomal storage diseases (LSD); evidence includes the presence of α-synuclein inclusions in the brain regions of patients with LSD and the identification of several lysosomal genes involved in LSD as genetic risk factors to develop PD.

## 1. Introduction

Lysosomes are subcellular acidic vesicles containing acid hydrolases. The main function of these organelles is the degradation of intracellular and extracellular macromolecules that are broken down into their primary constituents to be further recycled in the cytosol with the aim of building new cellular components.

The cell relies on lysosomes to properly eliminate and recycle proteins, lipids, polysaccharides, carbohydrates, and other type of molecules to maintain cellular homeostasis. When the lysosome is unable to degrade these macromolecules, the abnormal accumulation of material within lysosomes first causes lysosomal impairment, which can ultimately lead to general cellular dysfunction.

To date, approximately seventy lysosomal storage diseases (LSDs) have been described, many of which are caused by loss-of function mutations in lysosomal hydrolases but can also be caused by other lysosomal-related proteins, such as lysosomal transmembrane proteins and members of the trafficking machinery that transport newly synthesized lysosomal proteins to the lysosomes [1].The main characteristic of LSDs is the accumulation of an indigestible substrate inside the lysosome that leads to general lysosomal dysfunction and consequently to cellular and tissue damage. Several LSDs exhibit neurodegeneration as one of their main features [2]. In addition, neurodegenerative diseases such as Parkinson’s disease (PD), Alzheimer’s disease (AD), Huntington’s disease (HD), amyotrophic lateral sclerosis (ALS), and several others have lysosomal dysfunction as one of the molecular pathways involved in their etiology and development. This bidirectional link between neurodegeneration and lysosomal dysfunction emphasizes the importance of lysosomal function in maintaining neuronal homeostasis.

## 2. The Autophagy–Lysosomal–Endosomal System

Neurons, due to their morphology, metabolic features, and postmitotic state, are particularly vulnerable to deregulation of the clearance systems that are needed to provide new components to build macromolecules as well as to eliminating toxic or unwanted components.

**Figure 1:**
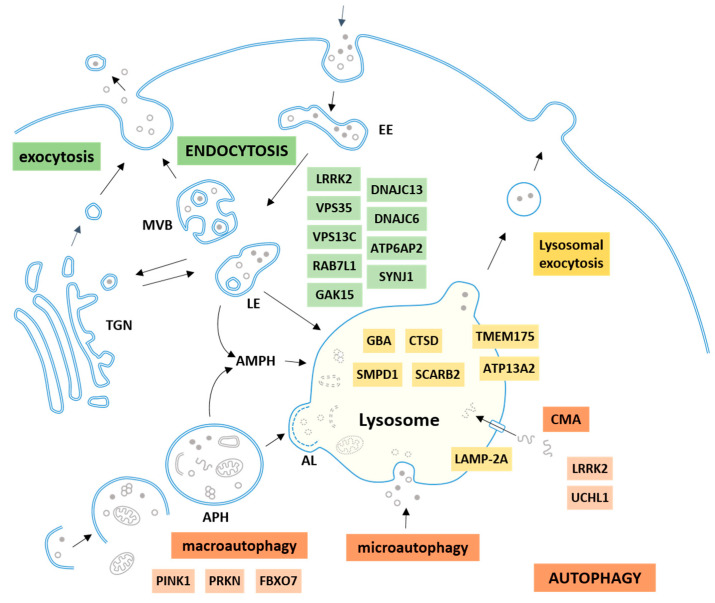
The autophagy–lysosomal–endosomal system and the Parkinson’s disease (PD)-related proteins. Lysosomes are the terminal compartment where the intracellular macromolecules delivered by autophagy (orange) and the extracellular macromolecules delivered by endocytosis (green) are degraded. Three types of autophagy are described in mammalian cells: macroautophagy, microautophagy, and chaperone-mediated autophagy (CMA). Macroautophagy eliminates intracellular components including proteins and organelles; these substrates are enclosed in a double membrane vesicle called an autophagosome (APH) that can be directly fused with lysosomes forming an autolysosome (AL). Alternatively, an APH can also first fuse with a late endosome (LE) to form an amphisome (AMPH) before fusing with the lysosome in order to degrade the cargo. Extracellular macromolecules and plasma membrane components can also be eliminated inside the lysosomes by endocytosis. Following the internalization, cargo is transported to early endosomes (EEs), the initial sorting station in the endocytic pathway; then EEs can be sorted into different routes, recycled back to the cell surface, enter the retrograde pathway to be sent to the trans-Golgi network (TGN), or maturate into multivesicular bodies (MVB) or late endosomes (LE), which enter the degradation pathway, and finally merge with the lysosome. PD-related proteins are listed alongside the pathway or organelle in which they are involved.

Lysosomes are the terminal compartment where intracellular macromolecules delivered by autophagy and extracellular macromolecules delivered by endocytosis are finally degraded. The whole system, known as the autophagy–lysosomal–endosomal system, is a complex network of coordinated and connected mechanisms with the lysosome as the terminal point and where the different pathways converge (Figure 1).

Lysosomes are loaded with approximately 60 different types of hydrolytic enzymes capable of degrading all types of diverse macromolecules such as proteins, polysaccharides, lipids, carbohydrates, and nucleic acids. Once macromolecules are broken down, their essential components are transported to the cytosol for their reuse or alternatively to be released from the cell through exocytosis. The role of lysosomes in the degradation and recycling of extracellular and intracellular components is a key factor in the maintenance of cellular homeostasis [3,4]

### 2.1. Autophagy

Autophagy, from the Greek “auto” (self) and “phagy” (eating), is the catabolic process by which intracellular components are delivered to lysosomes for degradation. Delivery of cytosolic material to lysosomes in mammalian cells can occur through three different routes that differ in the specificity of the target material to be degraded (cargo), how it is delivered into lysosomes and the mechanisms of regulation [5,6].

Macroautophagy, usually referred to only as “autophagy”, is responsible for the degradation of most intracellular components as a major mechanism to maintain cellular homeostasis and the proper balance between protein synthesis and degradation. It consists of the sequestration of cargo within double membrane vesicles called autophagosomes that will fuse to lysosomes for degradation of their contents. Macroautophagy is considered a dynamic process that comprises the sequential steps of initiation, elongation of the autophagosome membrane, recognition and trapping of the cargo, transport of the loaded autophagosomes towards the lysosomes, fusion of the autophagosome with lysosome, and, ultimately, degradation of the cargo inside the lysosomes (Figure 1). 

Macroautophagy substrates can include nonspecific cytosolic content or selective cargo that are recognized and delivered to lysosomes through highly regulated mechanisms. These forms of selective macroautophagy include the degradation of mitochondria (mitophagy) [7], peroxisomes (pexophagy) [8], endoplasmic reticulum (ER) portions (reticulophagy) [9], ribosomes (ribophagy) [10], lipid droplets (lipophagy) [11], pathogens (xenophagy) [12], and ubiquitinated-aggregates (aggrephagy) [13], among others. 

Basal macroautophagy is responsible for the continuous turnover of intracellular components, while induced autophagy is a stress-response mechanism activated under different conditions such as starvation, exposure to oxidative stress, hypoxia, or mitochondrial damage. Thus, autophagy can be activated under situations where specific materials such as misfolded or aggregated proteins, damaged organelles, or external pathogens must be eliminated from the cell or when essential basic elements such as amino acids are needed to rebuild new macromolecules. Mammalian target of rapamycin complex I (mTORC1) is the main negative regulator of macroautophagy and induces autophagy in response to amino acid deprivation (starvation), insulin, growth factors, ATP levels, glucose, hypoxia, and other forms of stress [14].

Chaperone-mediated autophagy (CMA) is the second type of autophagy in mammalian cells. In this pathway, substrates are soluble cytosolic proteins containing a specific CMA motif related to the pentapeptide KFERQ [15]. In the cytosol, substrate proteins are recruited by the chaperone complex through the direct interaction of the CMA motif of the substrate protein and the Hsc70 chaperone. Next, the substrate–chaperone complex is transported to the lysosomal surface and interacts with the multimeric lysosome-associated membrane protein 2 (LAMP-2A) translocation complex. Translocation of the substrate protein across the LAMP-2A complex takes place, and once the substrate protein is inside the lysosomal lumen, it is rapidly degraded, while the LAMP-2A translocation complex is dissembled into monomers until the next substrate–chaperone complex binds LAMP-2A again [16]. The high selectivity of CMA allows a timed degradation of specific proteins.

Finally, microautophagy is the third type of known autophagy and is a relatively poorly studied mechanism in mammalian cells that involves the sequestration and degradation of complete regions of the cytosol, including proteins and organelles, through the direct invagination of the lysosomal membrane itself [17].

In the central nervous system (CNS), the autophagy pathways are particularly relevant since most of the cells are postmitotic, which requires good quality control systems to eliminate altered proteins and damaged organelles [18]. In fact, it has been demonstrated that deletion of crucial macroautophagic genes in the mouse brain results in neurodegeneration secondary to the progressive accumulation of misfolded aggregated proteins and dysfunctional organelles [19,20]. Several neurodegenerative diseases, and Parkinson’s disease in particular, have been exhibit to present autophagy dysfunction, and this impairment may have an important role in the pathogenesis of these disorders. 

### 2.2. Endocytosis

Endocytosis is the process by which the cell internalizes proteins and lipids from the plasma membrane and extracellular material [21]. After internalization through different routes, cargo is delivered in early endosomes. Once there, cargo can be recycled back to the plasma membrane directly or through recycling endosomes [22]. Altmaon withernatively, cargo can be retained in early endosomes that will maturate into late endosomes through changes in luminal pH, activation and recruitment of distinct Rab guanosine triphosphates (RAB GTPases), and alteration in key phosphatidylinositol lipids [23]. Ultimately, late endosomes will fuse to lysosomes where cargo can be degraded. During the process, there is also a constant trafficking of material between endosomes and the trans-Golgi network (Figure 1).

Endocytosis has a special relevance in the brain, given that it is involved in essential functions such as neurotransmitter and neurotrophic signaling [24,25]. Since neuronal cells are highly polarized and are larger in size in comparison to other cell types, they require a highly specialized and complex endocytic machinery [26]. Alterations in this complex system have also been described in PD, suggesting the major role of this pathway in the pathogenesis of the disorder.

## 3. Parkinson’s Disease and Alpha-Synuclein

Parkinson’s disease is the second most common neurodegenerative disorder, after Alzheimer’s disease, and the most common movement disorder. It affects 1% of the population over the age of 60 [27,28]. The onset of the disease occurs normally at 65–70 years, with earlier cases usually linked to genetics. Clinically, it is characterized by a triad of motor symptoms, namely resting tremor, rigidity, and bradykinesia; supporting nonmotor symptoms; and neuropsychiatric alterations, autonomic dysfunction, sleep disorders, and hyposmia, among others [27,29,30].

The main neuropathological feature is the death of dopaminergic neurons from the substantia nigra pars compacta (SNpc), which explains the motor symptoms, and the presence of intracytoplasmic protein inclusions called Lewy bodies in the affected areas [31].

PD can be classified as familial or sporadic according to the presence or absence of a family history of PD. Approximately 15% of the cases are considered familial; however, only approximately 5–10% present a monogenic form of the disorder. Several genes have been associated with both autosomal dominant and recessive forms of PD, including *SNCA*, *LRRK2*, *PRKN* (*PARK2*), *PINK1*, *PARK7* (*DJ-1*), *VPS35*, *ATP13A2*, *PLA2G6*, and *FBXO7* [32,33]. In addition, other genes such as *TMEM230*, *LRP10*, *NUS1*, and *ARSA* have recently been postulated as disease-causing candidates in Mendelian PD; however, further studies are needed to confirm their involvement in the development of the disorder [32].

In sporadic cases, the etiology is multifactorial and involves an interplay between environmental and genetic factors. The main risk factor for PD is age [34]; however, increasing evidence indicates a major contribution of genetics. Candidate genes and genome-wide association studies (GWAS) have allowed the identification of a large number of risk genes and loci for sporadic PD (sPD) [35,36,37,38]. To date, mutations in the *GBA* gene, which encodes the lysosomal enzyme β-glucocerebrosidase (GCase), are the major genetic risk factors for PD that have been described [39,40]. Other well-known candidate risk PD genes are *LRRK2*, *SNCA*, and *MAPT* [36], and some of these genes (*LRRK2* and *SNCA*) have been associated with both Mendelian and sporadic forms of PD, indicating that different disease-related genetic mechanisms may coexist at the same locus [41].

Interestingly, an important number of these risk factors and identified causal genes for monogenic forms of PD are involved in the autophagy–lysosomal–endosomal pathways, pointing to the major involvement of these pathways in the pathogenesis of the disorder.

α-Synuclein protein is the main component of Lewy bodies found in the affected brain areas of PD patients. Accordingly, PD is considered a synucleinopathy that encompasses neurodegenerative diseases characterized by the progressive accumulation of α-synuclein aggregates in the CNS. In addition to PD, dementia with Lewy bodies (DLB), and multiple system atrophy (MSA) are also synucleinpathies that accumulate cytoplasmic synuclein aggregates in the frontal cortex and inside oligodendrocytes, respectively [42].

α-Synuclein is a small (14 kDa) soluble protein. In humans, it is encoded by the *SNCA* gene, and was the gene where the first autosomal dominant mutations for PD were found [43,44,45]. In neurons, α-synuclein is mainly localized at the presynaptic terminal and is involved in the regulation of synaptic activity, plasticity, synaptic vesicle pool maintenance and trafficking [43,44,45], although its full role is still not completely understood. It has three functional domains: an N-terminal domain, a central non-amyloid-component (NAC) domain, and a C-terminal tail. The central NAC domain is enriched in hydrophobic residues and is prone to oligomerization [46,47,48]. The N-terminal domain is rich in lysine residues and can form amphipathic α-helices for lipid interaction, which are important for membrane interactions. Interestingly, PD-linked disease mutations are found in the amphipathic region, and it is known that sphingolipid metabolites that accumulate in LSDs can interact with α-synuclein and induce its aggregation, which suggests that the interaction between α-synuclein and lipids plays an important role in PD pathogenesis and partially explains the selectivity of α-synuclein aggregation when lipid degradation is altered [28,47,48].

## 4. Lysosomal-Dependent Degradation of Synuclein

Alpha-synuclein is found abundantly in physiological conditions. Normal levels of α-synuclein inside the cell are maintained through the balance between synthesis and degradation. Given that the oligomeric state of α-synuclein as a monomeric, oligomeric, fibrillary, or aggregated protein is a process highly dependent on protein concentration, an alteration in any of the mechanisms regulating a-synuclein turnover leads to an increased cytoplasmic concentration of α-synuclein and consequently promotes the formation of oligomeric and fibrillary species [28]. The presence of α-synuclein oligomers and protofibrils is postulated to be toxic for neurons, suggesting that the accumulation of α-synuclein is an important mechanism in the degeneration of dopaminergic neurons that occurs in PD [48].

Increased expression of α-synuclein caused by multiplications (duplications or triplications) of the *SNCA* gene [49,50,51], as well as polymorphisms in the promoter region [52,53], increases α-synuclein levels and can lead to accumulation inside neurons. In a similar manner, point mutations in the *SNCA* gene [43,44,45,49,54,55,56] can impair its degradation, contributing to α-synuclein accumulation [57]. In addition to these genetic variants observed in dominant familial forms of PD or parkinsonian syndromes, large-scale genetic studies have also identified polymorphisms in the *SNCA* gene that constitute risk factors for sPD [36].

Since altered levels of synuclein can promote PD progression, α-synuclein turnover mechanisms play an important role in PD development, and autophagy pathways have a key role in maintaining proper α-synuclein neuronal levels. Wild-type α-synuclein is mostly degraded through the autophagic pathways—macroautophagy but predominantly CMA [57,58,59,60]. In addition, and like many other cytosolic proteins, α-synuclein can also be degraded simultaneously by the ubiquitin-proteasome system (UPS), although it is not the main mechanism, as inhibition of proteasome function does not result in abnormal accumulation of α-synuclein [59]. In contrast, inhibiting either CMA or macroautophagy leads to α-synuclein accumulation [58]. Consequently, it is expected that alterations in the lysosomal function might disturb α-synuclein turnover and promote increasing α-synuclein levels.

α-Synuclein contains the CMA-motif [57], which is recognized by the chaperone HSC70; the protein–chaperone complex is recruited to the lysosomal membrane and interacts with the CMA receptor LAMP-2A, and α-synuclein is translocated into the lysosomal lumen to be degraded [57,58,61]. However, alterations in α-synuclein protein due to a mutation or posttranslational modifications can affect the turnover of α-synuclein by CMA. Some mutant forms of α-synuclein that are linked to familial cases of PD (p.A30P and p.A53T) are not efficiently degraded through CMA; these mutant α-synucleins can bind LAMP-2A on the lysosomal surface with high affinity but are not internalized inside the lysosomes, preventing its own degradation and furthermore blocking CMA-dependent degradation of other substrates [57,58,62,63]. In addition to these rare α-synuclein mutations, other posttranslational modifications of α-synuclein that have been described in PD patients, including phosphorylated, nitrated, oxidized, oligomeric, and dopamine-modified α-synuclein forms, also present altered CMA-degradation rates [63]. The decrease in the capacity of these α-synuclein species to be translocated and eliminated through the CMA pathway favors the increase of these soluble forms in the cytosol and promotes the formation of oligomeric protofibril intermediates, which usually progress to insoluble α-synuclein fibrils.

Consistent with these findings, in vitro and in vivo studies have confirmed the key role of CMA in α-synuclein turnover and intracellular levels [58,61,62,64], and decreased levels of CMA markers have been reported in postmortem nigral samples from PD patients [65,66].

Taken into account the key role of the lysosomal-dependent degradation of α-synuclein and the consequences of its inefficient turnover, we can assume that direct or indirect alteration of the lysosomal function, but also the autophagic or endosomal machinery, can trigger the abnormal accumulation of α-synuclein. In the following sections, we will discuss the contribution of these dysfunctions to the pathogenesis of PD.

## 5. Lysosomal Dysfunction in Parkinson’s Disease

The capacity for lysosomal processing diminishes progressively with aging [67], and as mentioned previously, multiple lines of evidence indicates that lysosomal dysfunction is implicated in several neurodegenerative disorders including PD, AD, ALS, frontotemporal dementia (FTD) and HD, among others [68,69].

The relevance of lysosomal dysfunction in the pathogenicity of PD has been supported by evidence coming from genetic studies (Table 1). For instance, pathogenic variants in *GBA* confer a high risk of developing PD, and mutations in the *ATP13A2* gene, which encodes for a lysosomal ATPase, cause a juvenile-onset familial form of parkinsonism [70]. In recent years, GWAS have also identified several candidate genes that encode lysosomal proteins including *TMEM175*, *CTSB*, *SCARB2*, *ATP6V0A1*, *GALC*, *GUSB*, and *NEU1* as risk factors for PD [36,37,38,71,72]. Furthermore, using whole-exome sequencing, which allows the identification of rare disease-associated coding variants that are not captured by GWAS, Robak and colleagues observed that 56% of individuals with PD had at least one rare putative damaging variant in genes related to LSDs [73]. This excessive burden of rare variants in LSD genes in PD patients was confirmed in two independent cohorts, and researchers identified the lysosomal genes *CTSD*, *SLC17A5*, and *ASAH1* as new risk factors for PD. However, confirmation of these variants by other studies with larger data sets is required [73]. Recently, another study analyzed rare variants in genes related to lysosomal function [74]. An association of PD with the presence of rare variants in the lysosomal genes *GBA*, *ATP13A2*, *LAMP1*, and *TMEM175* was reported. Altogether, this evidence points towards a model in which common and rare variants in lysosomal genes may contribute to lysosomal dysfunction influencing PD susceptibility. Other factors that may affect lysosomal function indirectly and may modify the risk to develop PD are noncoding variants, copy-number variants, epigenetic factors, and gene–gene and gene–environment interactions. Although few studies have addressed these factors, there is preclinical evidence that, for instance, gene–environment interactions occur [75].

Below we describe some of these lysosomal proteins that have been linked to PD.

*GBA* encodes for the lysosomal enzyme β-glucocerebrosidase (GCase), which hydrolyses glucosylceramide (GlcCer) to ceramide and glucose. Homozygous and heterozygous compound mutations in the *GBA* gene cause Gaucher’s disease (GD), which is one of the most common LSD. It is characterized by a decrease in GCase activity and the subsequent accumulation of glucosylceramide in several organs. GD patients but also *GBA* asymptomatic carriers have a five-fold increase in their lifetime risk of developing PD in comparison to the general population [40]. In fact, mutations in the *GBA* gene are the most common genetic risk factor for PD [39,40]. Similar to idiopathic PD, the neuropathological hallmarks found in GBA-PD patients include loss of nigral dopamine neurons and the presence of Lewy bodies and neurites [76]. However, they manifest an earlier onset, worse and faster progression of motor symptoms, greater cognitive decline, and increased risk of mortality [76,98,99,100,101]

The mechanisms by which decreased GCase activity contributes to the pathogenesis of PD are not completely understood; however, a relationship between GCase alterations and α-synuclein pathology has been well established [102,103]. Thus, *GBA* mutations or a reduction in GCase activity through pharmacological or genetic approaches have resulted in the accumulation of α-synuclein in several in vivo and in vitro models, similar to what is observed in PD-GBA brains [104,105,106,107,108,109].

Two main theories have been postulated linking GCase with α-synuclein, namely loss-of-function and toxic gain-of-function of GCase activity [110]. The loss-of-function hypothesis states that GCase depletion leads to substrate accumulation, causing alterations in glycosphingolipid homeostasis and lysosomal dysfunction, which ultimately affects α-synuclein trafficking, processing and clearance. This model is sustained because all *GBA* mutations that cause increased risk of PD produce a reduction in GCase activity. The other hypothesis states that toxicity may occur through a gain-of-function mechanism; misfolded GCase proteins are retained in the ER, overwhelming the folding machinery, and promoting ER stress [111] and α-synuclein accumulation [112]. It has also been described that the accumulated α-synuclein may impair trafficking of GCase from the ER to the lysosomes, resulting in a positive feedback loop propagating the disorder [104]. Both hypotheses are not mutually exclusive, and it is likely that a combination of both has a role in α-synuclein and PD pathogenesis. In addition, several toxic mechanisms have been described in nonneuronal models of mutant *GBA* [113,114,115], indicating that cell death may also occur due to events independent of α-synuclein accumulation.

In patients without *GBA* mutations, decreased enzymatic activity of GCase was also found, particularly in the SNpc [116,117,118] but also in other regions such as the anterior cingulate cortex, caudate, hippocampus, and cerebellum, with some discrepancies between studies [104,116,117,118,119,120]. It could be hypothesized that neurodegeneration, which appears in most of these brain regions in PD, may contribute to decreased levels of GCase activity; however, Murphy and colleagues [119] detected a loss of GCase activity in lysosomal-enriched protein fractions of the anterior cingulated cortex of early PD patients prior to neuronal loss. In addition, the cerebellum is not one of the main regions involved in the neurodegenerative PD process [116]. As a final point, GCase activity declines progressively during the aging of healthy individuals [120], which may contribute in part to identifying aging as the major risk for developing PD.

The *ATP13A2* gene encodes for a lysosomal transmembrane ATPase pump that is involved in cation and polyamine transport, the polyamine-transporting ATPase 13A (ATP13A2) [121]. Mutations in this gene cause Kufor–Rakeb syndrome, an autosomal recessive atypical form of early-onset parkinsonism [70]. Moreover, a specific homozygous mutation (p.M810R) in *ATP13A2* has been reported to cause an LSD, a form of juvenile neuronal ceroid lipofuscinosis [122].

In sPD, some studies have linked heterozygous variants in the *ATP13A2* gene to early-onset PD [123,124,125,126], whereas others have not found an association [127,128,129]. A recent study linked rare variants in this gene to PD in a large cohort (4096 PD patients, mean age of onset 60 yr., and 4096 controls), supporting that *ATP13A2* mutations are a risk factor for PD [74]. Changes in ATP13A2 mRNA and protein levels have been detected in dopaminergic SN neurons of sPD patients [70,130,131], further supporting the involvement of ATP13A2 in sporadic forms of PD.

Patient-derived fibroblasts from patients with Kufor–Rakeb syndrome showed alterations in lysosomal function, including impaired acidification and decreased proteolytic processing causing insufficient substrate degradation and autophagosome clearance [132,133]. While in vitro studies have established that ATP13A2 deficiency leads to α-synuclein accumulation, contradictory results have been observed in mouse models. Whereas some authors found brain accumulation of α-synuclein in *Atp13a2*-null mice, others found no changes [134,135]. In a recent study using double mutant mice, *Atp13a2*-null mice overexpressing human synuclein showed a summatory effect of both genotypes in the progression of sensorimotor deficits, supporting the pathological interaction between both loci [136]. Analysis of postmortem brain samples of Kufor-Rakeb syndrome patients may help to elucidate whether mutations in *ATP13A2* cause this atypical form of PD through a mechanism dependent on α-synuclein pathology. Defects in mitochondria and clearance of divalent metals secondary to ATP13A2 deficiency may also play a role [137]. How PD heterozygous variants in *ATP13A2* affects the activity of the protein and how this affects the functionality of these processes should also be studied in detail.

The *TMEM175* gene encodes a transmembrane protein, the endosomal/lysosomal potassium channel TMEM175, which is a K^+^ channel located in late endosomes and lysosomes. As described previously, this gene has been proposed as a candidate PD susceptibility gene by GWAS and DNA sequencing studies [36,37,74]. Jinn and colleagues demonstrated that deficiency of *TMEM175* in a neuroblastoma model resulted in lysosomal and mitochondrial dysfunction, providing functional evidence of the role of this protein in the pathogenesis of PD [138]. Interestingly, knock-out of TMEM175 in neuroblastoma cells resulted in decreased GCase activity. Furthermore, knockdown of TMEM175 in rat primary hippocampal neurons exposed to exogenous α-synuclein fibrils showed increased phosphorylated α-synuclein aggregation, indicating that this protein may be linked to these two well-known PD risk factors [138].

The *SMPD1* gene encodes the lysosomal enzyme sphingomyelin phosphodiesterase/acid sphingomyelinase (ASMase), which hydrolyzes sphingomyelin into ceramide and phosphocholine. Biallelic mutations in this gene cause the LSD Niemann–Pick diseases types A and B [139]. Loss-of-function of ASMase leads to the accumulation of sphingomyelin and its deacetylated form lyso-sphingomyelin [84,140] within the lysosome in different organs. Type A patients present progressive neurodegeneration and short life expectancy, whereas type B patients do not generally show signs of CNS involvement.

It has been reported in multiple studies that *SMPD1* carriers present a higher risk of developing PD than the normal population [85,141,142,143]. A recent study demonstrated that p.L302P and p.fsP330 mutations, which cause Niemann–Pick type A and have also been associated with PD, impair the trafficking of ASMase to the lysosome in cellular models. In addition, deficiency of SMPD1 resulted in increased α-synuclein levels [86]. Further studies should elucidate whether reduced SMPD1 lysosomal localization increases α-synuclein levels contributing to PD pathogenesis.

The *CTSD* gene encodes a relevant lysosomal enzyme, cathepsin D, the main aspartic endoprotease responsible for the degradation of long-lived proteins. Biallelic mutations in the *CTSD* gene cause a severe neurodegenerative LSD known as neuronal ceroid lipofuscinosis 10 (CLN10) [87]. In addition, cathepsin D has been related to PD; on the one hand, *CTSD* has been proposed by Robak and colleagues [74] as a candidate PD susceptibility gene, although a later study did not replicate the result [74]. On the other hand, different studies have reported alterations in the activity and expression of this protein in PD individuals. A decrease in cathepsin D activity was reported in the temporal cortex and frontal cortex (trend) of late-stage sPD patients [117,144]. Reduced expression of its protein levels was also found in SN and was particularly extended in α-synuclein inclusions [145]. In contrast, others reported no changes in SN [116] or even an increase in the anterior cingulate cortex in early-stage PD patients [119].

It is important to note that cathepsin D is involved in the degradation of monomeric and aggregated α-synuclein [146,147]. In fact, cathepsin D deficiency leads to intracellular accumulation of this protein in dopaminergic cells, and mice and infant brains of patients with neuronal ceroid lipofuscinoses [148,149]. A recent study described that GBA mutations may increase monomeric α-synuclein levels by decreasing cathepsin D levels in dopaminergic neurons [150]. Further studies should explore whether in PD patients and changes in cathepsin D in specific brain regions play a role in α-synuclein accumulation and to what extent these variations are mediated by decreased GCase activity.

The *GLA* gene encodes α-galactosidase A. Mutations in this gene cause Fabry disease, an X-linked LSD in which the decrease in α-galactosidase activity leads to accumulation of glycosphingolipids, including globotriaosylceramide (Gb_3_) and globotriaosylsphingosine (lyso-Gb_3_), with a main effect in the vascular endothelium. An association between Fabry disorder and PD has been proposed, since some Fabry patients present clinical history of parkinsonism [151]. In addition, an online survey suggested that Fabry patients and their relatives present an increased risk of developing PD [152].

Besides, in SN and temporal cortex of sPD patients, α-galactosidase activity was shown to be reduced [144,153]. Interestingly, the decrease of α-galactosidase activity in the temporal cortex correlated with an increase in α-synuclein phosphorylated at serine 129, pointing to a link between both phenomena [144]. This was supported by α-galactosidase-deficient mice in which brain accumulation of phosphorylated α-synuclein, concomitant with disruption of the autophagic–lysosomal pathway, was also reported [154]. In addition, a recent report described the presence of α-synuclein-immunopositive Lewy pathology in a 58-year-old Fabry patient [92]. All this evidence suggests the possible role of α-galactosidase in the pathogenicity of PD linked to α-synuclein accumulation; however, the possible underlying mechanism remains unknown.

Evidence of lysosomal dysfunction also comes from the analysis of postmortem brain samples of PD individuals. In the SN, lysosomal depletion and decreased levels of different lysosomal-associated proteins, including LAMP-2A, lysosomal-associated membrane protein 1 (LAMP-1), and heat shock cognate 71 kDa protein (HSC70), have been observed [65,145,155]. Furthermore, as described above, alterations in the concentration and activity of several lysosomal enzymes including GCase, cathepsin D and α-galactosidase, in brain samples of sPD have been described [116,117,144,153]. In contrast, most authors did not find changes in the activity of other enzymes, such as β-galactosidase, β-hexosaminidase and cathepsin B and E [116,117,118,144], which suggests that the dysfunction inside lysosomes is selective. However, Huebecker and colleagues recently found a decrease in the activity of β-galactosidase and β-hexosaminidase in SN of two cohorts of patients, highlighting discrepancies among different studies [153]. Discrepancies may be due to differences in age of the subjects, stage of PD, postmortem delay, or processing of the samples. It may be interesting to assess the activities of these lysosomal enzymes in enriched-lysosomal fractions, given that subtle differences missed sometimes in total homogenate fractions can be revealed when specific subfractions are analyzed.

## 6. Lysosomal Storage Disorders and Parkinson’s Disease

In addition to the implication of lysosomal proteins in PD pathogenesis, a bidirectional link between LDSs and PD has been established. As described above, a higher burden of rare variants in LDS genes has been reported in PD patients [73]. Furthermore,, different evidence has linked LSDs with PD: (1) neurodegeneration is one of the main features of LDSs [2]; (2) parkinsonism has been reported in some patients with different LSDs [151,156,157]; (3) an increased risk of PD has been well established in Gaucher’s disease [39,40], and it has been suggested in Fabry disease [152]; and (4) aggregation of α-synuclein has been reported in the brain of patients and animal models of different LSDs, including Gaucher’s disease [158], Fabry disease [92], Niemann-Pick disease [159,160], Sandhoff disease, Tay-Sachs disease, metachromatic leukodystrophy, β-galactosialidosis and GM2 gangliosidosis [161], Krabbe disease [162], and neuronal ceroid-lipofuscinosis [163].

Despite this evidence, in addition to Gaucher disease, which is one of the most prevalent LSD [164], few patients have been diagnosed with both PD and LSDs. Some of the reasons may be the low prevalence of LSDs, the underdiagnoses of LSDs, and the fact that patients with severe infantile forms die within the first years of life, which might be not enough time to develop PD symptoms.

Most of the lysosomal genes proposed as a genetic risk factors for PD cause a LSD when biallelic mutations are present (Table 1). Moreover, these biallelic variants may also increase the risk to develop PD. Given that most LSDs are recessive, it can be hypothesized that dysfunction of proteins and the subsequent accumulation of substrates may lead to lysosomal dysfunction, contributing to the development of PD through different mechanisms including α-synuclein accumulation. In carriers, monoallelic mutations may affect the function of the protein by haploinsufficiency or dominant effects, and although whether substrate accumulation occurs is not clear [165,166], other mechanisms, such as changes in the activity of cathepsins, may produce defects in lysosomal functioning [150]. One of the hypotheses is that the general impairment of lysosomal function that seems to occur over the lifespan may be exacerbated in carriers of specific variants in lysosomal genes [167]. It is likely that each of the variants in each of the LSD genes leads to lysosomal dysfunction preferentially through different pathogenic pathways, which may determine their contribution to increasing the risk to develop PD.

## 7. Autophagy Dysfunction in Parkinson’s Disease

As mentioned above, an important link between disruptions in the autophagic pathways (both CMA and macroautophagy) and the pathogenesis of PD has been observed, since alterations in these systems have been observed in familial and sporadic cases of the disease. Interestingly, mutations in some genes involved in the autophagy machinery have been identified as mutations with Mendelian inheritance or risk factors in PD.

As previously mentioned, CMA impairment due to the presence of mutant or modified α-synuclein species can play an important role in PD etiology. In addition to α-synuclein genetic and posttranslational modifications, other PD-related proteins have been shown to affect autophagy activity [57,63].

Leucine-rich repeat serine/threonine-protein kinase 2(LRRK2) is a large protein with multiple protein–protein interaction domains and two enzymatic regions with GTPase and kinase activity. Due to its ability to interact with several proteins, LRRK2 is a dynamic protein capable of forming different complexes and controlling diverse functions at different subcellular locations, cell types, and conditions and accordingly has been implicated in a large number of cellular pathways [168]. Therefore, mutations in this gene have been implicated in multiple pathogenic mechanisms in PD, including the dysfunction of the autophagic–lysosomal–endosomal system in parallel to other cellular mechanisms such as the inflammatory response, oxidative stress, mitochondrial dysfunction, and synaptic dysfunction.

Mutations in *LRKK2* are the most known genetic cause of familial PD, accounting for 5% of total familial cases [169,170]. Although many *LRRK2* mutations have been described, only a few have been proven to cause PD, and p.G2019S is the most abundant. As also observed in other PD-related genes, GWAS analysis has also identified genetic variants at the *LRRK2* locus as risk factors for sPD [35,171].

Several studies have shown that mutant *LRRK2* can interfere with macroautophagy activity, although the exact mechanism that explains how LRRK2 regulates the macroautophagy pathway is still not clear and remains controversial [172].

In addition to the role of LRRK2 in macroautophagy, mutant forms of the protein have also been involved in CMA inhibition. LRRK2 is a CMA substrate; however, the p.G2019S LRRK2 mutated protein remains stacked in the CMA receptor on the surface of the lysosomes, avoiding its own degradation and inhibiting the degradation of other CMA substrates, including α-synuclein, which tends to oligomerize into toxic species [173].

Ubiquitin carboxyl-terminal hydrolase isozyme L1 (UCHL1), a ubiquitin hydrolase associated with familial PD, also behaves like mutant α-synuclein and mutant LRRK2; it interacts with the lysosomal membrane receptor LAMP-2A to be digested through the CMA pathway, but in its mutated form it remains stacked in the lysosomal membrane and blocks CMA machinery [174]. Although LRKK2 and UCHL1 are not directly related to the CMA machinery, when mutated, they can impair CMA, and both are an examples that highlight the importance of α-synuclein turnover and the pathogenic consequences of its accumulation due to problems in its degradation by the CMA pathway.

*PINK1* and *PRKN* are two genes linked to familial cases of PD. Their products, PINK1 and E3 ubiquitin–protein ligase Parkin, are involved in mitophagy; the selective recognition and degradation of mitochondria by macroautophagy [175].

PINK1 is important as a stress sensor for mitochondria. It is present at low levels on healthy mitochondria, as it is rapidly degraded as soon as it reaches the mitochondria. However, in damaged or depolarized mitochondria, the degradation process of PINK1 is incomplete, and the full-length protein accumulates on the outer mitochondrial membrane (OMM), where it can phosphorylate different proteins, including Parkin and ubiquitin (UB). Parkin is an E3 ubiquitin ligase and requires a phosphorylation mediated by PINK1 for its activation [176,177]. Active Parkin ubiquitinates a variety of substrates present on the OMM, either elongating pre-existing ubiquitin chains or ubiquitinating substrates de novo. These phosphorylated polyUB chains on the mitochondrial surface are the signal to recruit mitophagy receptors and promote the interaction between damaged mitochondria and the nascent autophagosome [178,179,180]. Accordingly, Parkin-mediated ubiquitination together with PINK1-mediated phosphorylation of UB and Parkin feeds a positive feedback loop, amplifying the “eat-me” signals that lead to mitochondrial degradation [181].

In terms of genetics, *PRKN* is the most common autosomal recessive PD-causing gene, especially relevant in juvenile-onset cases [60,182]. It usually causes symmetrical parkinsonism with slow progression. The physiopathological alterations are variable, and some cases have been reported without Lewy body pathology [182], suggesting that the dysfunction it causes is not directly linked to α-synuclein accumulation. Most of its mutations are found in the ubiquitin-like domain [29]. *PINK1* causes early-onset autosomal recessive PD and accounts for 1–9% of familial cases. Most of its mutations affect the kinase domain, causing a loss-of-function [29], but do not affect its mitochondrial localization [182].

Mutations in *PINK1* and *PRKN* genes involved in the initiation of mitophagy can affect the efficiency of this pathway and lead to the inaccurate turnover of mitochondria, accumulation of damaged mitochondria, and ultimately impairment of the bioenergetics of the cell. The presence of mutations in these genes highlights the link between autophagy/mitophagy activity and mitochondrial dysfunction in the pathogenesis of PD [183].

Additionally, PINK1 and Parkin perform alternative roles in the mitochondrial quality control system of neurons beyond mitophagy and act as stress sensors of different pathways such as immune response. Neuroinflammation has been described to contribute to neuronal degeneration in PD and other neurodegenerative diseases [184]. Both PINK1 and Parkin have an influence in these processes, specifically in the innate immune response. PINK1 upregulates the IL-1β-mediated signaling cascade at several steps, increasing signalosome formation. Therefore, its alteration impacts glial innate immune responses and increases neuroinflammation [185,186]. Parkin is also involved in innate immune response through abnormal NLRP3 inflammasome activation [187]. Alteration of both genes causes defects in immune response and enhances neuronal death.

Another gene related to both PD and mitophagy is *FBXO7*. Mutations in this gene cause autosomal recessive PD or a parkinsonism disorder, sharing some characteristics with *PRKN*-associated PD [188,189]. The protein is part of an E3 ubiquitin–protein–ligase complex that mediates the phosphorylation-dependent ubiquitination of proteins, targeting them for proteasomal degradation [190]. FBXO7 also participates in mitophagy by targeting Parkin to depolarized mitochondria after PINK1 phosphorylation of ubiquitin [191,192]; therefore, mutations in this gene cause deficiencies in Parkin recruitment and mitophagy initiation. In *Drosophila*, the mutant Parkin phenotype is rescued by overexpression of wild-type FBXO7 [193].

## 8. Endocytosis Dysfunction in Parkinson’s Disease

The endocytic system is composed of a series of highly dynamic networks of membrane-enclosed structures and includes different pathways to degrade, recycle, and sort proteins and lipids between the plasma membrane and intracellular compartments. The regulation of the endocytic network is mediated by several protein complexes and by small GTPases, the Rab proteins, and membrane-associated proteins that act as molecular switches to control the identity and relationship of the different types of endosomes [194]. We describe below different components and regulators of the endosome trafficking machinery that have been associated with familial forms of PD including Vacuolar protein sorting-associated protein 35 (VPS35), some Rab proteins, LRRK2, DnaJ homolog subfamily C member 13 (DNAJC13), Synaptojanin-1 (SYNJ1), and ATPase H(+)-transporting accessory protein 2 (ATP6AP2).

VPS35 is a part of the retromer complex involved in endosomal retrograde transport of vesicles to the trans-Golgi network (TGN) and the plasma membrane [195]. Following entry, cargo is transported to early endosomes (EE), the initial sorting station in the endocytic pathway; then, EE can be sorted into different routes, recycled back to the cell surface through recycling endosomes (RE), enter the retrograde pathway mediated by the retromer to be sent to the trans-Golgi network (TGN), or alternatively enter the degradation pathway through the maturation of the EE into late endosomes (LE), and finally be degraded in lysosomes [196]. The VPS35 protein is a core subunit of the retromer, and, together with vacuolar protein sorting-associated protein 29 (VPS29) and Vacuolar protein sorting-associated protein 26 (VPS26), it forms a complex implicated in cargo sorting and membrane tabulation [197]. Several retromer cargo proteins, such as the mannose-6-phosphate receptor, are essential for the transport and delivery of most of the lysosomal enzymes from the Golgi to their final destination in the lysosome; therefore, retromer dysfunction can lead to the disruption of lysosomal trafficking and functionality [198]. VPS35 also acts also as a scaffold for the binding of other protein complexes such as the WASP and Scar homologue (WASH) complex that regulates the endosomal trafficking machinery, the sorting of the endosomal cargo to appropriate destinations and ultimately autophagy [199].

Autosomal dominant mutations in the *VPS35* gene have connected retromer dysfunction with familial PD, although mutations in VPS35 are rare and account for only approximately 1% of familial parkinsonism [200,201]. The p.D620N mutation is the most common variant in VPS35-associated PD that clinically presents a symptomatology similar to idiopathic PD and a good response to levodopa therapy [202].

Several other endosomal proteins are involved in PD; DNAJC13, Putative tyrosine-protein phosphatase auxilin (DNAJC6), and the Rab protein GAK15 are involved in the uncoating of clathrin-coated vesicles [38,203,204], Ras-related protein Rab-7L1 has important roles in the retrograde trafficking of M6PRs to the Golgi apparatus [71,205,206], ATP6AP2 is essential for the biogenesis of active vacuolar H^+^-ATPase (V-ATPase) for the acidifications of LE and lysosomes [207], and SYNJ1 has an important role in endolysosomal trafficking of synaptic proteins [208,209].

Finally, the *LRRK2* gene, in addition to its role in autophagy, has also been associated with endocytosis. Among LRRK2 interactions, the protein can bind and phosphorylate proteins involved in the endocytosis machinery, such as VPS35 [210], VPS52 [211], and several Rab proteins [77,78,79,212,213]. Thus, mutations in *LRRK2* can also promote dysfunction of the endosomal system along with the effect of mutant LRRK2 protein in several other cellular processes.

Taken together, these findings point towards a defective endocytic system in several familial and sPD cases that may lead to lysosomal dysfunction and defects in proteostasis [80,81].

## 9. Role of Glial Cells in PD

Since the main hallmark of PD is the loss of dopaminergic neurons, most studies have focused on neuronal death. However, in recent years, emerging evidence points out that microglial and astrocytic dysfunction may also play an important role in the pathogenesis of PD.

Recently, it has been demonstrated that several genes that have been linked to PD are also expressed in glial cells and in some cases with comparable or even higher levels than neurons [82,83]. Remarkably, some of these genes play a role in astrocyte and microglial pathways including inflammatory response, immune signaling, oxidative stress, glutamate intake, lysosomal and mitochondrial function, and autophagy [83,88].

It is known that pathologic α-synuclein is released to neurons and can be internalized and degraded by astrocytes and microglia [89,90]. Interestingly, *GBA* and *LRKK2* mutations seem to impair lysosomal degradation of proteins in glial cells, contributing to further accumulation of α-synuclein [91,93]. This α-synuclein accumulation may compromise neuronal survival through α-synuclein glia-to-neuron transfer, which has already been demonstrated to occur in LRKK2 induced pluritpotent stem cells (iPSC)-derived astrocytes [93], or through inflammatory mechanisms [91,94]. Perturbations in other PD-associated genes such as *PINK1*, *PRKN*, and *PARK7* have also been described to alter different glial processes that can impact neuronal survival, including astrocyte proliferation, production of ROS, inflammatory responses, and mitochondrial function [95,96,97,214].

In summary, several PD-linked genes are also expressed in glial cells, and variants in these genes may affect different biological processes essential for CNS homeostasis, contributing to the pathogenesis and progression of PD.

## 10. Conclusions

In conclusion, multiple type of evidence support the major role of lysosomal dysfunction in the pathogenesis of PD, and also increasing data has linked LSDs with PD. Several genes involved in the lysosomal function, the autophagic pathways, and the endocytic network have been described to be causative in Mendelian forms of PD or have been suggested as a candidate risk of PD genes. A relationship between some of these genes and α-synuclein has been established suggesting that α-synuclein accumulation is secondary to lysosomal dysfunction and may lead to the development of PD. However, the mechanistic association between the pathogenic variants in most of the genes and α-synuclein accumulation remains to be established. The key role of the lysosome in PD has also opened therapeutic opportunities in the treatment of PD; to the well-known pharmacological autophagy inducers tested in the last decade to improve the macroautophagy activity in neurons as a common therapeutic strategy in neurodegenerative diseases [175], we can currently add a new approach with the lysosome as a target organelle. At present, different therapies approved or validated for the treatment of LSD are also being tested as possible therapies in PD [215,216,217], opening new opportunities in the disease modifying therapies in PD.

## Figures and Tables

**Table 1:** Lysosomal genes associated with PD.

Gene	Protein	Function	LSD	Type	Evidence	Ref.
***GBA***	Lysosomal acid GCase	Hydrolysis of glucosylceramide into ceramide and glucose	Gaucher disease	Risk factor	+++	[36,37,38,39,75,76,77]
***ATP13A2***	ATPase cation transporting 13A2	Cation transporter and polyamine exporter	Neuronal ceroid lipofuscinosis	Risk factor/AR (Kufor Rakeb)	+++	[76,78,79,80,81,82,83]
***TMEM175***	Endosomal/lysosomal potassium channel TMEM175	Conductance of potassium in lysosomes and endosomes		Risk factor	++	[36,37,38,76,84]
***SMPD1***	Sphingomyelin phosphodiesterase	Hydrolysis of sphingomyelin into ceramide	Niemann–Pick disease type A/B	Risk factor	++	[75,85,86,87,88]
***SCARB2***	Lysosome membrane protein 2	Receptor for lysosomal mannose-6-phosphate-independent targeting of GCase	Action myoclonus-renal failure syndrome	Risk factor	++	[38,61,74,89,90,91]
***CTSD***	Cathepsin D	Main aspartyl protease of the lysosome	Neuronal ceroid lipofuscinosis	Risk factor	+	[75]
***GLA***	α-galactosidase A	Hydrolysis the terminal alpha-galactosyl moieties from glycolipids and glycoproteins	Fabry disease	Risk factor	+	[92]
***CTSB***	Cathepsin B	Lysosomal cysteine protease		Risk factor	+	[36,37]
***GALC***	Galacto-cerebrosidase	Hydrolysis of galactose ester bonds from glycolipids	Krabbe disease	Risk factor	+	[36,37]
***ATP6V0A1***	V-ATPase 116 kDa subunit a1	Subunit of a vacuolar ATPase that mediates acidification		Risk factor	+	[36]
***GUSB***	β-glucuronidase	Hydrolysis of glycosaminoglycans	Mucopolysaccharidosis type VII	Risk factor	+	[37]
***NEU1***	Sialidase-1	Hydrolysis of the terminal sialic acid residues from sialylated glyco-conjugates	Sialidosis	Risk factor	+	[37]
***SLC17A5***	Sialin	Free sialic acid exporter from lysosomes	Salla disease	Risk factor	+	[75]
***ASAH1***	Acid ceramidase	Hydrolysis of sphingolipid ceramides into sphingosine and free fatty acids	Farber Lipogranulomatosis	Risk factor	+	[75]
***LAMP1***	Lysosome-associated membrane glycoprotein 1	Trafficking of cholesterol and lipids		Risk factor	+	[76]
***ARSA***	Arylsulfatase A	Hydrolysis of cerebroside sulfate to cerebroside and sulfate	Metachromatic leukodystrophy	Risk factor/AR	+	[75,93,94]
***NPC1***	NPC intracellular cholesterol transporter 1	Intracellular trafficking of cholesterol and lipids	Niemann-Pick type C	Risk factor	+	[95,96]
***NAGLU***	α-*N*-acetyl glucosaminidase	Hydrolysis of terminal *N*-acetyl-d-glucosamine residues in *N*-acetyl-α-d-glucosaminides	San Filippo syndrome B	Risk factor	+	[37,97]
